# 1687. Unprecedented Outbreak of Invasive group A *Streptococcus* Infections in Children - Colorado, October 2022 to March 2023

**DOI:** 10.1093/ofid/ofad500.1520

**Published:** 2023-11-27

**Authors:** Erin C Ho, Lori Silveira, Meghan C Birkholz, Jessica R Cataldi, Samuel R Dominguez

**Affiliations:** University of Colorado School of Medicine, Aurora, CO; University of Colorado Anschutz Campus, Aurora, Colorado; Children's Hospital Colorado, Clifton, Virginia; University of Colorado School of Medicine, Aurora, CO; University of Colorado School of Medicine, Aurora, CO

## Abstract

**Background:**

Since the fall of 2022, we have observed a sharp rise in pediatric invasive group A *Streptococcus* (iGAS) hospitalizations in Colorado, similar to trends reported in other countries.

**Methods:**

From October 2022 – March 2023, iGAS cases were prospectively identified in patients hospitalized at Children’s Hospital Colorado and medical charts were systematically reviewed. iGAS was defined as isolation of GAS from a sterile site (confirmed case) or a non-sterile site with clinically consistent disease (probable). Using laboratory specimen records, we compared the number of patients with sterile site GAS-positive cultures across three time periods: Pre-COVID-19 (Jan 2015 – Mar 2020), COVID-19 pandemic (Apr 2020 – Sep 2022), and Outbreak (Oct 2022 – Mar 2023).

**Results:**

Among the 81 iGAS cases identified during the outbreak **(Table 1)**, median age was 6 years old; 64% of patients were male and 69% were previously healthy. 33% of patients required pediatric intensive care unit (PICU) admission, and two patients died. iGAS was more commonly associated with upper respiratory infection (URI) symptoms (63%) than isolated sore throat (5%) or trauma/skin findings (15%). The most common clinical manifestations were head and neck infections (37%), musculoskeletal infections (32%) and pneumonia (20%). 11% had toxic shock syndrome and 4% had necrotizing fasciitis. Lab findings associated with PICU admission were bandemia, leukopenia and higher CRP values. Disease severity and treatment varied by clinical manifestation **(Table 2)**. There were more iGAS cases during the outbreak (average 10.0/month) compared to pre-pandemic years (3.8/month over the same period) and during the pandemic (1.2/month over the same period) **(Figure 1)**.

**Figure:**
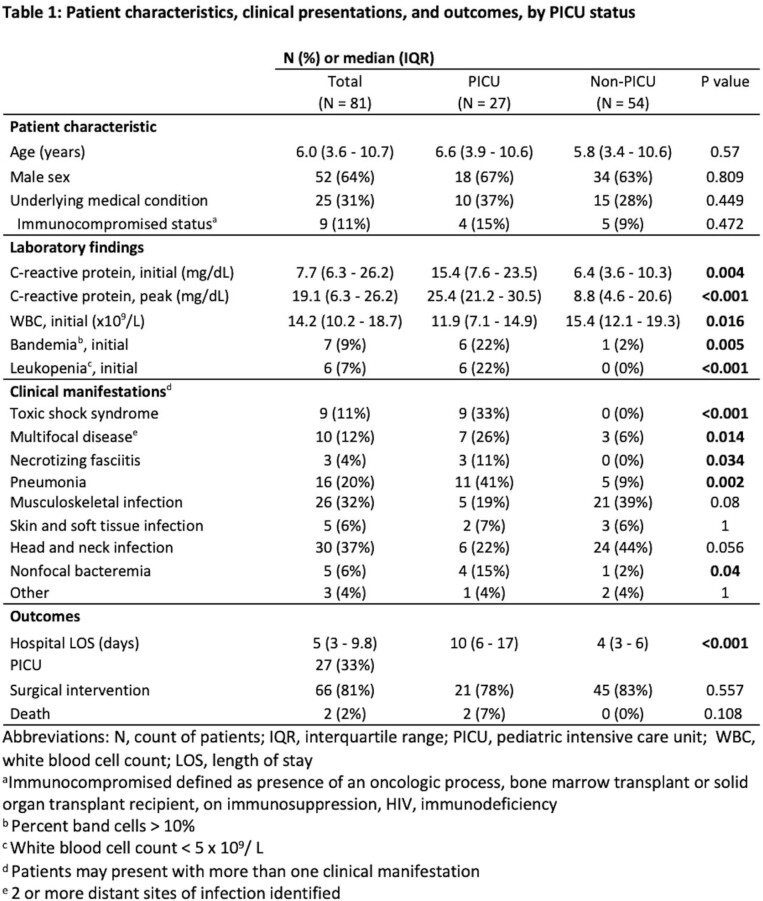

**Figure:**
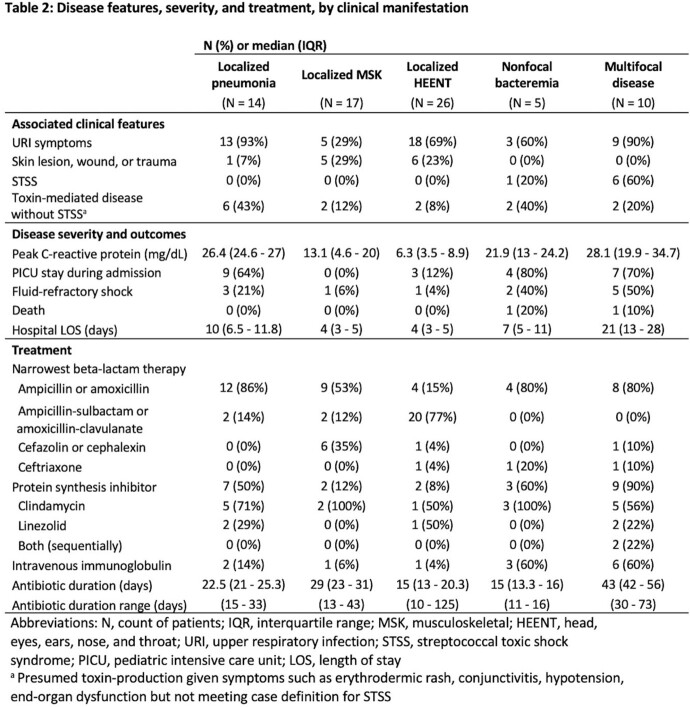

**Figure:**
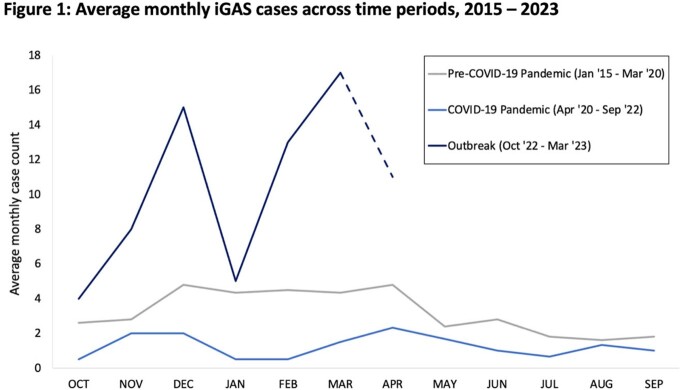

**Conclusion:**

We report an unprecedented outbreak of iGAS in pediatric patients in Colorado, with case numbers close to triple the pre-pandemic baseline at our hospital. The timing and high proportion of cases with associated URI symptoms suggest a link to the recent surges in respiratory viruses, although iGAS cases have continued to persist past the peak of respiratory season. Invasive GAS can be severe and evolve rapidly; there are important clinical and laboratory features that may help in earlier identification of children who are critically ill.

**Disclosures:**

**Samuel R. Dominguez, MD, PhD**, Biofire Diagnostics: Advisor/Consultant|Biofire Diagnostics: Grant/Research Support|Cobio Diagnostics: Board Member|Karius: Advisor/Consultant|Pfizer: Grant/Research Support

